# Incompatibilities in Prescriptions

**Published:** 1898-03

**Authors:** 


INCOMPATIBILITIES IN PRESCRIPTIONS. For students in pharmacy and medicine and practising pharmacists and physicians. By EDSEL A. RUDDIMAN, Ph. M., M. D., Adjunct Professor of Pharmacy and Materia Medica in Vanderbilt University. Octavo, pp. 267. New York: John Wiley & Sons; London: Chapman & Hall, Limited. 1897.

This work affords an excellent guide to the physician in prescription writing. To remember all incompatibilities is impossible, but the book is so conveniently arranged that it is but a minute's work to turn to the drug whose behavior in the combination is in doubt and find its reaction with various antagonistic substances clearly stated. The second half of the book is of interest to the student of pharmacy rather than to the medical man. It contains the formulæ of upwards of 300 prescriptions containing incompatibilities, which the author advises the student to study and discover for himself. Following this list are notes explanatory of the errors in the foregoing prescriptions, with suggestions to the druggist as to how many of the undesirable reactions may be avoided. Students in pharmacy and medicine, and the practising pharmacist and physician will all agree as to the excellence of this volume. M. J. F.

